# Supplementary material for: Lung extracellular matrix modulates KRT5+ basal cell activity in pulmonary fibrosis
Source: Nat Commun. 2023 Sep 27;14:6039. doi: 10.1038/s41467-023-41621-y (PMC10533905; doi:10.1038/s41467-023-41621-y)
Supplement: Supplementary file 1 — Supplementary Information [file 41467_2023_41621_MOESM1_ESM.pdf]

# Supplementary Information

## Lung extracellular matrix modulates KRT5+ basal cell activity in pulmonary fibrosis

Hewitt RJ[1,2], Puttur F[1], Gaboriau DCA[3], Fercoq F[4], Fresquet M[5], Traves WJ[1], Yates LY[1], Walker SA[1], Molyneaux PL[1,2], Kemp SV[2,6], Nicholson AG[2], Rice A[2], Roberts E[4], Lennon R[5], Carlin LM[4,7], Byrne AJ[1], Maher TM[1,8], Lloyd CM[1*].

**Affiliations:**

[1]National Heart and Lung Institute, Imperial College London, SW7 2AZ, UK.

[2]Royal Brompton and Harefield Hospitals, Guy's and St Thomas' NHS Foundation Trust, London, SW3 6NP, UK.

[3]Facility for Imaging by Light Microscopy, National Heart and Lung Institute, Imperial College London, SW7 2AZ, UK.

[4]Cancer Research UK Beatson Institute, Glasgow, G61 1BD, UK.

[5]Wellcome Centre for Cell-Matrix Research, Division of Cell-Matrix Biology and Regenerative Medicine, School of Biological Sciences, Faculty of Biology Medicine and Health, The University of Manchester, Manchester, M13 9PT, UK.

[6]Current address: Department of Respiratory Medicine, Nottingham University Hospitals NHS Trust, City Campus, Hucknall Road, Nottingham, NG5 1PB, UK.

[7]School of Cancer Sciences, University of Glasgow, Glasgow, G61 1QH, UK.

[8]Current address: Keck Medicine of USC, 1510 San Pablo Street, Los Angeles, California, 90033, USA.

***Corresponding author**: Clare M. Lloyd, National Heart and Lung Institute, Imperial College London, London, SW7 2AZ, UK. E-mail: c.lloyd@imperial.ac.uk

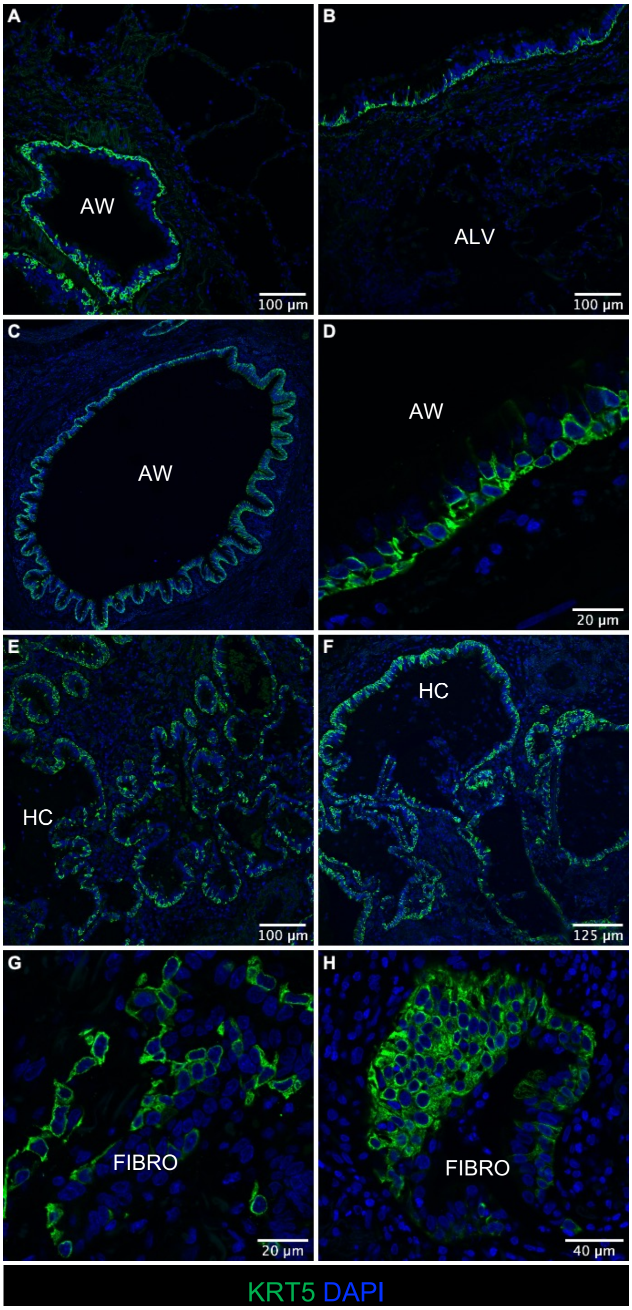


**Supplementary Fig. 1 | KRT5⁺ BC distribution in the normal and fibrotic distal lung. a – h** Distal lung tissue sections from controls and IPF patients immunostained for basal cell marker KRT5⁺ (green) and DAPI (blue). **a,b** In control lung tissue KRT5+ cells are found restricted to the distal airways (AW) and are absent from the alveolar tissue (ALV). **c,d** In IPF lung tissue, KRT5⁺ cells are again seen in the distal airways in a typical configuration but are also located in **e,f** honeycomb cysts (HC), and **g,h** fibrotic interstitium (FIBRO). **i** Overview of normal distal lung tissue showing KRT5⁺ cells (red) lining the airway but absent from the alveolar region. SHG signal (turquoise) in the peribronchial (PB) and perivascular (PVS) regions. Alpha-smooth muscle actin (yellow) outlining smooth muscle bands surrounding airways.

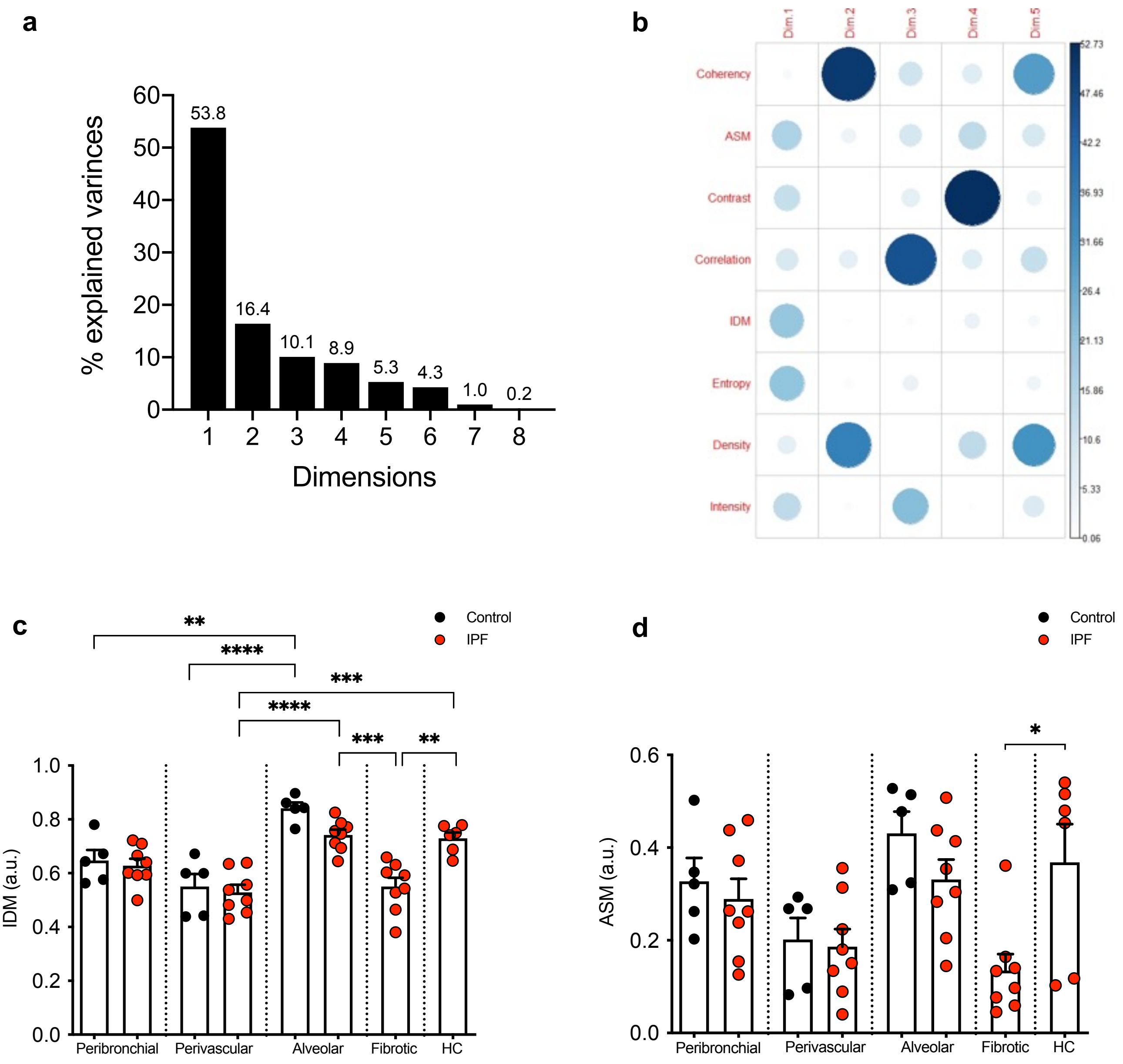


**Supplementary Fig. 2 | Quantitative image analysis of control and IPF distal lung. a** Scree plot of % variances explained by each component (dimension) of PCA plot shown in Fig. 1d. **b** Corr plot of variables contributing to each dimension of PCA. **c - d** Image texture analysis per region showing **c** inverse difference moment (IDM) and **d** angular second moment (ASM). Each data point represents the average of 2 – 6 images per area per control (*n* = 5) and IPF patient (*n* = 8 for PB, PVS, ALV, FIBRO and *n* = 6 for HC).

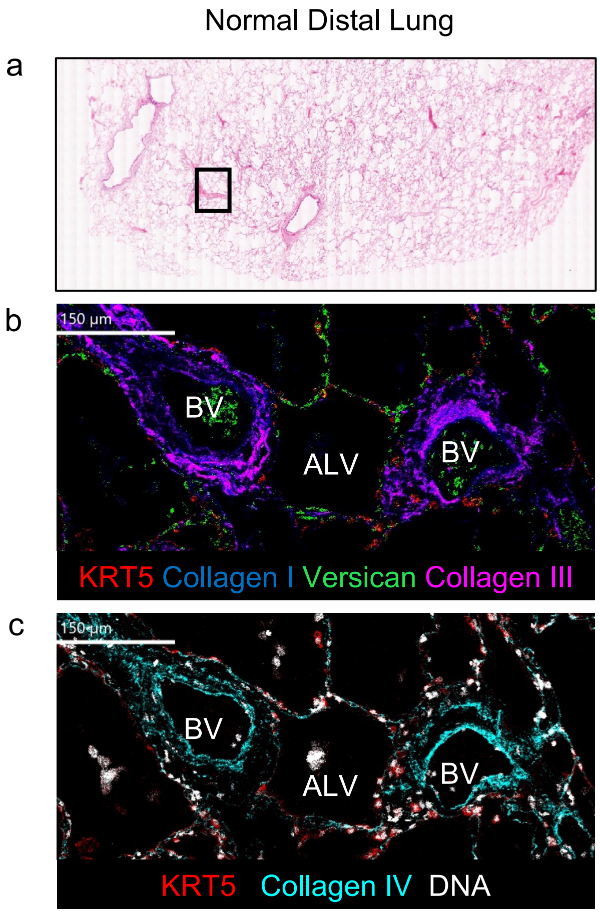


**Supplementary Fig. 3 | IMC of distal lung tissue from controls and patients with IPF** Imaging mass cytometry (IMC) of perivascular region of distal lung tissue from normal control ($n$ = 1). Representative image demonstrating key regions; blood vessel (BV) and alveolar (ALV). **a** H&E overview **b** distribution of ECM components; collagen I (blue), collagen III (magenta) and versican (green) in relation to $KRT5^+$ BCs (red) **c** collagen IV (turquoise) in relation to $KRT5^+$ BCs (red) and nuclear DNA marker (white).

**Supplementary Fig. 4. KRT5+ basal cells co-localise with fibroblasts in the fibrotic niche.**

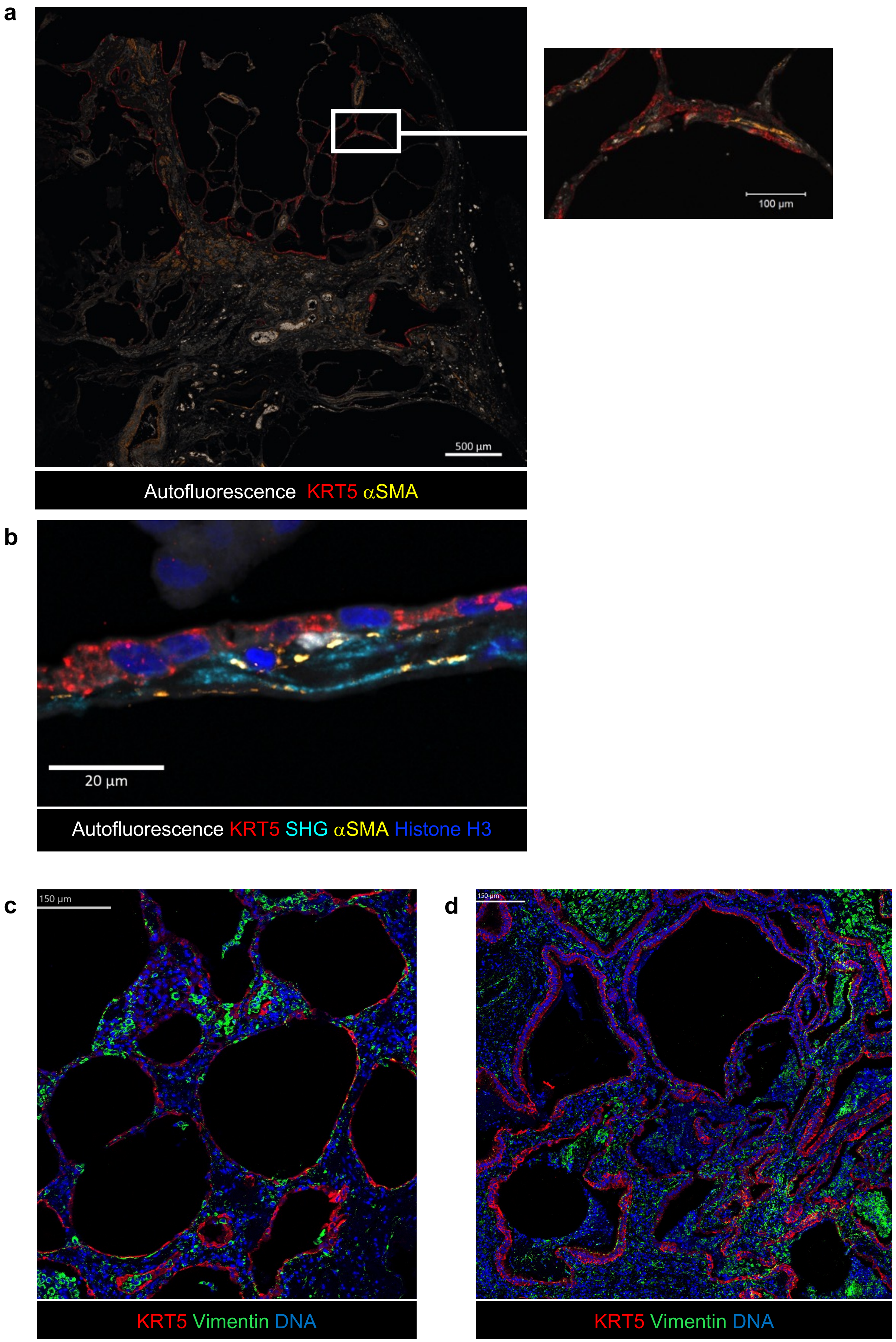

**Supplementary Fig.4 | KRT5+ basal cells co-localise with fibroblasts in the fibrotic niche. a** Overview of fibrotic, remodelled alveolar region of IPF lung with high-resolution image (white box) showing co-localisation of KRT5+ basal cells (red) with $\alpha$-SMA+ fibroblasts (yellow). **b** High resolution image of fibrotic alveolar region of IPF lung showing co-localisation of KRT5+ basal cells, $\alpha$-SMA+ fibroblasts and SHG signal (cyan). **c - d** Imaging mass cytometry of distal lung tissue from IPF patient showing KRT5+ BC (red), vimentin+ cells (green) and DNA (blue) in fibrotic alveolar region **c** and HC region **d**.

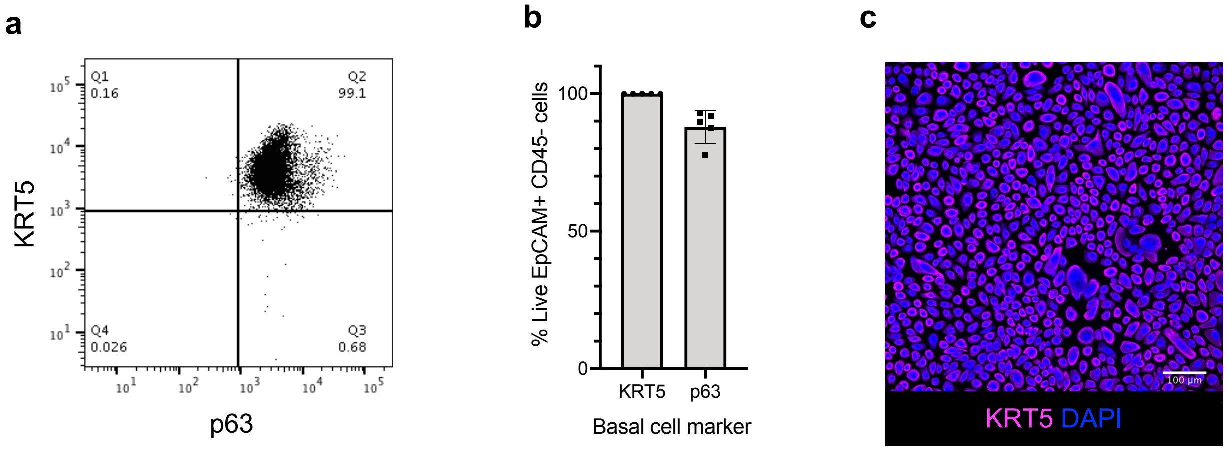


**Supplementary Fig.5 | Human airway basal cell phenotyping. a,b** Flow cytometry phenotyping of primary human airway epithelial cells (*n*=5) in submerged culture (passage 3) for basal cell markers KRT5 and p63. **c** Immunofluorescence microscopy for KRT5 in submerged culture. Representative image shown. Scale bar, 100µm.

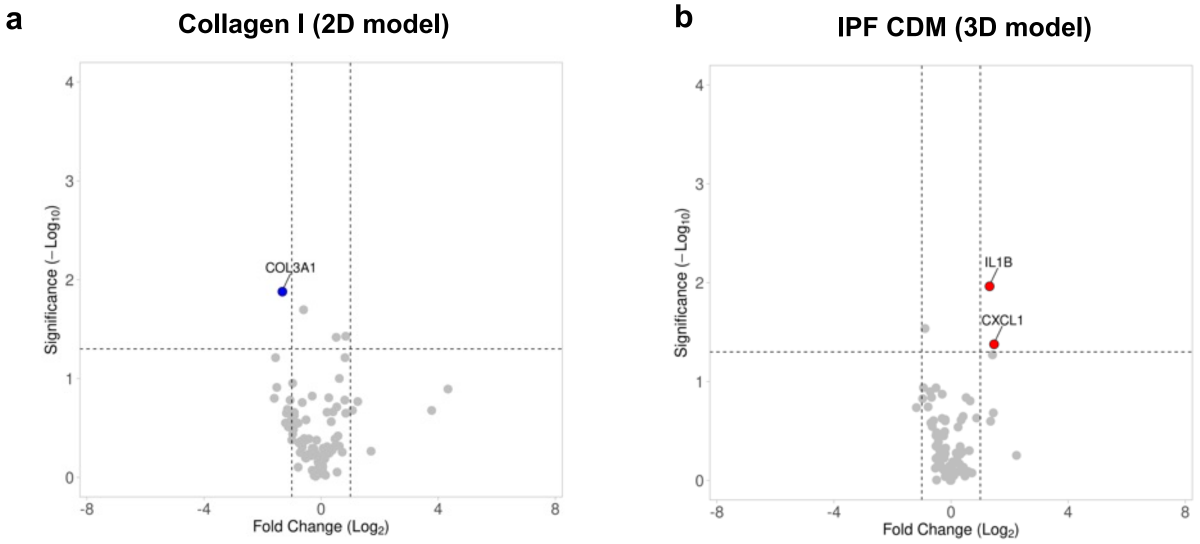


**Supplementary Fig.6 | Gene expression of IPF KRT5+ cells compared to healthy KRT5+ cells cultured on collagen I or IPF CDMs. a,b** Volcano plots showing gene expression changes determined by Qiagen $RT^2$ PCR Array between KRT5+ cells from IPF patients (*n* = 3) vs. healthy controls (*n* = 3) cultured on **a** collagen I or **b** IPF CDMs. Upregulated genes in IPF KRT5+ cells vs. control KRT5+ cells (red), downregulated genes in IPF KRT5+ cells vs. control KRT5+ cells (blue). Significance defined as log fold change >2 or <2, p<0.05. P values were calculated based on a Student's t-test of the replicate normalised gene expression values (2^(- Delta CT)) for each gene in control and test groups. Housekeeping genes used - ACTB, B2M and RPLP0.

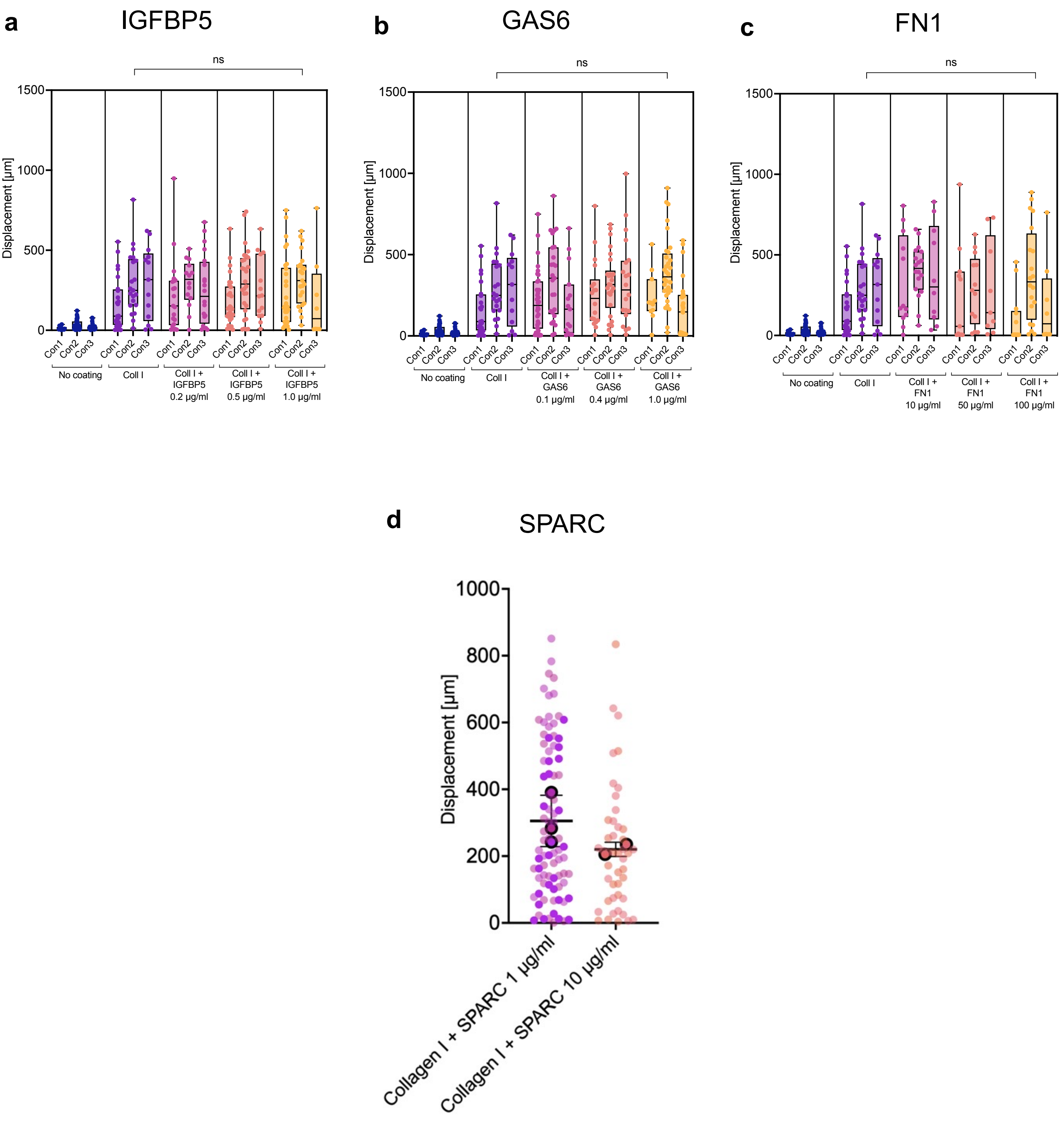


**Supplementary Fig.7 | Influence of proteins identified by MS-proteomics on KRT5⁺ cell migration. a – c** Box and whisker dot plots quantifying cell displacement per healthy control subject *(n = 3,* in duplicate*)*, over 12hrs for **a** IGFBP5; **b** GAS6 and **c** FN1. **d** Beeswarm SuperPlot showing displacement of cells over 12hrs for KRT5+ cells cultured on SPARC. Summary statistics with mean and standard deviation are superimposed on the plot. Sample-level means compared using a one-way ANOVA with Tukey's multiple comparison test.

**Supplementary Table S1 |** Demographic information for human lung tissue samples

| | Control (n = 5) | IPF (n = 8) |
|---|---|---|
| Age | 68 ± 4 | 65 ± 6 |
| Male / Female sex | 3 / 2 | 6 / 2 |

Age presented as mean ± standard deviation.

Control samples included normal parenchymal lung tissue obtained during resections for localised non-small cell lung cancer from sites remote to any tumour mass.

**Supplementary Table S2 |** Quantitative image analysis measurements.

| Measurement | Software | Description |
|---|---|---|
| Collagen intensity | Imaris Software, Oxford Instruments | Mean intensity of SHG signal in collagen fibres. |
| Collagen density | Imaris Software, Oxford Instruments | Collagen area represented as a percentage coverage of the total image area analysed. |
| Coherency | OrientationJ plugin, Fiji | Measures fibre orientation and coherency; a value of zero indicates perpendicular fibres, a value of 1 indicates parallel fibres. |
| Contrast | GLCM plugin, Fiji | Measures local variations in the grey-level co-occurrence matrix; a value of zero indicates a constant image with no variation. |
| Entropy | GLCM plugin, Fiji | Statistical measure of randomness, ranging from zero to infinity. |
| Correlation | GLCM plugin, Fiji | Linear dependency of grey levels on those of neighbouring pixels; higher values for similar grey-level regions. |
| Inverse Difference Moment (IDM) | GLCM plugin, Fiji | Local homogeneity or smoothness across an image; a value of 1 indicates a grey levels of the pixel pairs are similar |
| Angular Second Moment (ASM) | GLCM plugin, Fiji | Measures the number of repeated pixel pairs indicating uniformity of distribution of grey level in the image; a value of 1 indicates a constant image. |

GLCM; grey level co-occurrence matrix, SHG; second harmonic generation

**Supplementary Table S3 |** Demographic information for human KRT5+ BC and HLF samples

*KRT5+ basal cells - Fig. 3*

| | Healthy (*n* = 5) | IPF (*n* = 5) |
|---|---|---|
| Age | 63 ± 5 | 68 ± 10 |
| Male / Female sex | 4 / 1 | 4 / 1 |
| Smoking status | | |
| Current | 0 | 2 |
| Ex | 1 | 3 |
| Never | 2 | 0 |
| N/A | 2 | 0 |

*KRT5+ basal cells - Fig. 4*

| | Healthy (*n* = 4) | IPF (*n* = 4) |
|---|---|---|
| Age | 44 ± 12 | 76 ± 4 |
| Male / Female sex | 2 / 2 | 3 / 1 |
| Smoking status | | |
| Current | 0 | 0 |
| Ex | 3 | 1 |
| Never | 1 | 3 |
| N/A | 0 | 0 |

*KRT5+ basal cells - Fig. 5*

| | Healthy (*n* = 3) | IPF (*n* = 3) |
|---|---|---|
| Age | 47 ± 21 | 68 ± 14 |
| Male / Female sex | 2 / 1 | 3 / 0 |
| Smoking status | | |
| Current | 0 | 0 |
| Ex | 3 | 2 |
| Never | 0 | 1 |
| N/A | 0 | 0 |

*Human lung fibroblasts - Fig. 6*

| | Healthy (*n* = 3) | IPF (*n* = 3) |
|---|---|---|
| Age | 65 ± 1 | 60 ± 8 |
| Male / Female sex | 2 / 1 | 1 / 2 |
| Smoking status | | |
| Current | 0 | 0 |
| Ex | 1 | 0 |
| Never | 2 | 1 |
| N/A | 0 | 2 |

*KRT5+ basal cells - Fig. 7*

| | Healthy (*n* = 6) |
|---|---|
| Age | 56 ± 7 |
| Male / Female sex | 2 / 3 |
| Smoking status | |
| Current | 0 |
| Ex | 1 |
| Never | 4 |
| N/A | 1 |

Age presented as mean ± standard deviation.

**Supplementary Table S4 |** Antibodies used

| Primary antibodies used for immunofluorescence microscopy | Company | Catalog ID |
|---|---|---|
| Rabbit monoclonal to cytokeratin 5 (clone EP1601Y), AF 647 | Abcam | Cat# ab193895 |
| Rabbit polyclonal to cytokeratin 5 (clone Poly19055), unconjugated | BioLegend | Cat# 905501 |
| Rabbit polyclonal anti-collagen type I, unconjugated | Novus Biologicals | Cat# NB600-408 |
| Rabbit polyclonal anti-fibronectin, unconjugated | Abcam | Cat# ab2413 |
| Mouse monoclonal anti-fibronectin (clone FN-15), unconjugated | Sigma- Aldrich | Cat# F7387 |
| Mouse monoclonal to alpha-smooth muscle actin (clone 1A4), Cy3 | Sigma- Aldrich | Cat# C6198 |
| Mouse anti-histone H3 (C-terminus) (clone 1B1-B2), AF 594 | BioLegend | Cat# 819405 |
| **Primary antibodies used for flow cytometry** | | |
| Rabbit monoclonal anti-p63 (clone EPR5701), unconjugated | Abcam | Cat# ab124762 |
| Mouse monoclonal anti-human CD326/ EpCAM (clone 9C4), AF 488 | BioLegend | Cat# 324209 |
| Mouse monoclonal anti-human CD45 (clone H130), BV 605 | BioLegend | Cat# 304042 |
| Rabbit monoclonal to cytokeratin 5 (clone EP1601Y), AF 647 | Abcam | Cat# ab193895 |
| **Primary antibodies used for mass cytometry** | | |
| Rabbit monoclonal to cytokeratin 5 (clone EP1601Y), unconjugated | Abcam | Cat# ab52635 |
| Goat polyclonal anti-collagen type I – 169Tm | Standard BioTools Inc. | Cat# 3169023D |
| Rabbit polyclonal anti-collagen type III alpha 1, unconjugated | Novus Biologicals | Cat# NB600-594 |
| Rabbit polyclonal anti-collagen type IV, unconjugated | Novus Biologicals | Cat# NB120-6586 |
| Recombinant rabbit monoclonal anti-versican antibody (clone EPR12277), unconjugated | Abcam | Cat# ab240200 |
| Rabbit monoclonal anti-fibronectin 175Lu (clone EPR23110—46) | Standard BioTools Inc. | Cat# 91H034175 |
| Rabbit monoclonal anti-vimentin (clone D21H3) – 143Nd | Standard BioTools Inc. | Cat# 3143027D |
| **Secondary antibodies** | | |
| Goat anti-rabbit IgG (H+L), AF 680 | ThermoFisher | Cat# 10585543 |
| Goat anti-mouse IgG (H+L), DyLight 800 4X PEG Conjugate | Cell Signalling | Cat# 5257 |
| Goat anti-rabbit IgG, DyLight 488 | Invitrogen | Cat# 35552 |
| Goat anti-rabbit IgG, AF 647 | ThermoFisher | Cat# A-21244 |
| Goat anti-mouse IgG, AF 546 | Invitrogen | Cat# A-11030 |
| Goat anti-mouse IgG, AF 568 | Invitrogen | Cat# A-11004 |
| Goat Anti-Rabbit IgG, BV421 | BD Biosciences | Cat# 565014 |
