# Supplementary material for: Lung extracellular matrix modulates KRT5+ basal cell activity in pulmonary fibrosis
Source: Nat Commun. 2023 Sep 27;14:6039. doi: 10.1038/s41467-023-41621-y (PMC10533905; doi:10.1038/s41467-023-41621-y)
Supplement: Supplementary file 3 — Description of Additional Supplementary Files [file 41467_2023_41621_MOESM3_ESM.pdf]

Description of Additional Supplementary Files

File Name: Supplementary Data 1
Description: Mass spectrometry (MS)-based proteomics dataset. Tab 1 provides a list of all 2794 proteins identified by mass spectrometry. Tab 2 provides a list of 156 matrix proteins identified by cross-referencing to the human matrisome database. The remaining tabs provide the source data for each panel in Figure 6.

File Name: Supplementary Movie 1
Description: KRT5+ BCs tracked on ECM ligands, collagen I and versican over 12 hours. Images acquired on JuLI real-time cell history recorder and tracked using Nikon NIS- Elements software.

File Name: Supplementary Movie 2
Description: Fluorescently labelled KRT5+ BCs migrating through CDM tracked over 12 hours using Nikon NIS-Elements software. Images acquired on a Zeiss Axio Observer Z1 Inverted Widefield Microscope with Lumencor SpectraX LED illumination and Zen Blue software.

File Name: Supplementary Movie 3
Description: Fluorescently labelled KRT5+ BCs migrating through fibrotic CDMs over 12 hours. Images acquired on a Zeiss Axio Observer Z1 Inverted Widefield Microscope with Lumencor SpectraX LED illumination and Zen Blue software.

File Name: Supplementary Movie 4
Description: Fluorescently labelled KRT5+ BCs migrating through fibrotic CDMs over 12 hours. Images acquired on a Zeiss Axio Observer Z1 Inverted Widefield Microscope with Lumencor SpectraX LED illumination and Zen Blue software.
