# Supplementary material for: Lung extracellular matrix modulates KRT5+ basal cell activity in pulmonary fibrosis
Source: Nat Commun. 2023 Sep 27;14:6039. doi: 10.1038/s41467-023-41621-y (PMC10533905; doi:10.1038/s41467-023-41621-y)
Supplement: Supplementary file 9 — Reporting Summary [file 41467_2023_41621_MOESM9_ESM.pdf]

nature portfolio

Corresponding author(s): Professor Clare Lloyd

Last updated by author(s): 07/09/2023

Reporting Summary

Nature Portfolio wishes to improve the reproducibility of the work that we publish. This form provides structure for consistency and transparency in reporting. For further information on Nature Portfolio policies, see our Editorial Policies and the Editorial Policy Checklist.

Statistics

For all statistical analyses, confirm that the following items are present in the figure legend, table legend, main text, or Methods section.

n/a	Confirmed	
☐	☒	The exact sample size (*n*) for each experimental group/condition, given as a discrete number and unit of measurement
☐	☒	A statement on whether measurements were taken from distinct samples or whether the same sample was measured repeatedly
☐	☒	The statistical test(s) used AND whether they are one- or two-sided<br>*Only common tests should be described solely by name; describe more complex techniques in the Methods section.*
☒	☐	A description of all covariates tested
☐	☒	A description of any assumptions or corrections, such as tests of normality and adjustment for multiple comparisons
☐	☒	A full description of the statistical parameters including central tendency (e.g. means) or other basic estimates (e.g. regression coefficient) AND variation (e.g. standard deviation) or associated estimates of uncertainty (e.g. confidence intervals)
☐	☒	For null hypothesis testing, the test statistic (e.g. *F*, *t*, *r*) with confidence intervals, effect sizes, degrees of freedom and *P* value noted<br>*Give P values as exact values whenever suitable.*
☒	☐	For Bayesian analysis, information on the choice of priors and Markov chain Monte Carlo settings
☒	☐	For hierarchical and complex designs, identification of the appropriate level for tests and full reporting of outcomes
☐	☒	Estimates of effect sizes (e.g. Cohen's *d*, Pearson's *r*), indicating how they were calculated

Our web collection on statistics for biologists contains articles on many of the points above.

Software and code

Policy information about availability of computer code

Data collection

Immunofluorescence imaging of human lung tissue and cells:
- Leica Application Suite (LAS) X v3.5.7.23225
- ZEISS ZEN 2.3 (Black Edition)

Imaging mass cytometry (IMC) of human lung tissue:
- CyTOF Software v7.0, MCD viewer v1.0.560.6 (Standard BioTools Inc)

Cell migration experiments:
- JuLI Stage Real-Time Cell History Recorder
- LEICA LAS X v3.5.7.23225
- ZEISS ZEN 2.6 (Blue Edition)

Flow cytometry:
- BD LSR Fortessa III cell analyser

Data analysis

Immunofluorescence imaging processing/ analysis:
- Fiji/ ImageJ (http://fiji.sc) v1.51
- Icy (https://icy.bioimageanalysis.org/) v2.0.0.0

Image texture analysis:

- Imaris v9.6.0 (Oxford instruments)
- OrientationJ (http://bigwww.epfl.ch/demo/orientation/)
- GLCM plugins for Fiji/ ImageJ (https://imageJ.nih.gov/ij/plugins/texture.html)
- GraphPad Prism v9.4.1

Cell migration experiments:
- Nikon NIS Elements v.4.50
- GraphPad Prism v9.4.1
- Motility lab (http://www.motilitylab.net/)

Flow cytometry:
- FlowJo7 (BD Life Sciences)

Wound healing PCR array:
- GeneGlobe Data Analysis Center (Qiagen)
- ClustVis (https://biit.cs.ut.ee/clustvis/)
- VolcaNoseR (https://huygens.science.uva.nl/VolcaNoseR/)

Mass spectrometry proteomics:
- Proteome Discoverer v2.4.1.15 SP1 (PD) (Thermo Scientific)
- ClustVis (https://biit.cs.ut.ee/clustvis/)
- STRING v11 (https://string-db.org/)

Principal component analysis (PCA) plots were generated in RStudio using freely available code (CC BY-NC-SA 3.0 US) : http://www.sthda.com/english/articles/31-principal-component-methods-in-r-practical-guide/112-pca-principal-component-analysis-essentials/. The R script used can be found at https://doi.org/10.5281/zenodo.8288989. For image texture analysis, Fiji/ImageJ pluggins were used: Texture Analyzer (https://imagej.nih.gov/ij/plugins/texture.html) was used to perform GLCM analysis and OrientationJ (http://bigwww.epfl.ch/demo/orientation/#measure) was used to measure the orientation of collagen fibres. A Fiji/imageJ macro containing command lines is provided at https://doi.org/10.5281/zenodo.8288989.

For manuscripts utilizing custom algorithms or software that are central to the research but not yet described in published literature, software must be made available to editors and reviewers. We strongly encourage code deposition in a community repository (e.g. GitHub). See the Nature Portfolio guidelines for submitting code & software for further information.

Data

Policy information about availability of data

All manuscripts must include a data availability statement. This statement should provide the following information, where applicable:

- Accession codes, unique identifiers, or web links for publicly available datasets
- A description of any restrictions on data availability
- For clinical datasets or third party data, please ensure that the statement adheres to our policy

Source data are provided with this paper. The mass spectrometry proteomics data have been deposited to the ProteomeXchange Consortium via the PRIDE90 partner repository with the dataset identifier PXD037236. Raw image files are stored on servers at Imperial College London due to their large file size.

Research involving human participants, their data, or biological material

Policy information about studies with human participants or human data. See also policy information about sex, gender (identity/presentation), and sexual orientation and race, ethnicity and racism.

Reporting on sex and gender	The human participants providing samples for our study were broadly sex-matched and are detailed in Supplementary Tables 1 and 3. Our study was not sufficiently powered to detect differences according to sex.
Reporting on race, ethnicity, or other socially relevant groupings	We did not report on race, ethnicity, or other socially relevant groupings in this manuscript.
Population characteristics	Healthy subjects (n=10) and patients with IPF (n=10) were prospectively recruited at the Royal Brompton Hospital (London, UK) between March 2017 and March 2020. A diagnosis of IPF was made following multi-disciplinary discussion, according to international guidelines. Healthy control subjects included previous and non-smokers with normal lung function (FEV1 >80% predicted for age and height, FEV1/ FVC ratio >70%). All subjects underwent fibreoptic bronchoscopy in accordance with a standard operating procedure. Bronchial brushings were used to collect airway epithelial cells for culture and expansion of KRT5+ basal cells.<br><br>Fresh lung tissue specimens and archived formalin fixed paraffin embedded (FFPE) lung tissue sections were obtained from the Royal Brompton Hospital (London, UK). All specimens were reviewed by a consultant histopathologist. Normal parenchymal lung tissue (control tissue) was obtained during resections for localised lung cancers from sites remote to any tumour mass (fresh n = 3, FFPE n = 5). IPF tissue samples with evidence of a usual interstitial pneumonia (UIP) histological pattern were procured from explant lung tissue or surgical lung biopsies (fresh n=3, FFPE n = 8) . These samples were used for culturing primary lung fibroblasts and imaging.<br><br>Details of diagnosis, sex, age and smoking status are provided in Supplementary Tables 1 and 3.

Recruitment	Healthy control subjects and patients with IPF with prospectively recruited to the study.
Ethics oversight	Ethical approval was granted by the Research Ethics Committee (NRES reference: 15/SC/0101, 15/LO/1399, 15/SC/0569) at the Royal Brompton Hospital (London, UK). All human participants provided written, informed consent. This research was performed in accordance with the Declaration of Helsinki.

Note that full information on the approval of the study protocol must also be provided in the manuscript.

Field-specific reporting

Please select the one below that is the best fit for your research. If you are not sure, read the appropriate sections before making your selection.

☒ Life sciences ☐ Behavioural & social sciences ☐ Ecological, evolutionary & environmental sciences

For a reference copy of the document with all sections, see nature.com/documents/nr-reporting-summary-flat.pdf

Life sciences study design

All studies must disclose on these points even when the disclosure is negative.

Sample size	Sample size is indicated in the figure legend for each experiment. No a priori power calculation was performed. Primary human cells derived directly from patients and healthy volunteers are a valuable and limited resource and as such it was not possible to perform pilot experiments to guide a power calculation. In line with previously published studies using primary epithelial cells (PMID: 34315881, PMID: 36163190) we used >3 independent biological replicates for our in vitro experiments. Phenotypes were consistent across donors from each group tested therefore we concluded an adequate sample size had been achieved.
Data exclusions	No data exclusion
Replication	Biological replicates are indicated in each respective figure legend. Data was replicated across the samples tested. For image texture analysis (Fig. 1) each data point represents the average of 2 – 6 images per area per subject with IPF (n=8) and control (n= 4-5). For the cell migration experiments we have indicated the total number of individual cells tracked per group in the results section. For the CDM cell migration work, at least n>2 technical replicates per donor were used.
Randomization	KRT5+ BCs from healthy controls and IPF patients were randomly allocated to experiments according to the availability of samples.
Blinding	For the cell migration analysis the operator analysing the image was blinded and samples decoded after analysis

Reporting for specific materials, systems and methods

We require information from authors about some types of materials, experimental systems and methods used in many studies. Here, indicate whether each material, system or method listed is relevant to your study. If you are not sure if a list item applies to your research, read the appropriate section before selecting a response.

Materials & experimental systems

n/a	Involved in the study	
☐	☒	Antibodies
☐	☒	Eukaryotic cell lines
☒	☐	Palaeontology and archaeology
☒	☐	Animals and other organisms
☒	☐	Clinical data
☒	☐	Dual use research of concern
☒	☐	Plants

Methods

n/a	Involved in the study	
☒	☐	ChIP-seq
☐	☒	Flow cytometry
☒	☐	MRI-based neuroimaging

Antibodies

Antibodies used

Primary antibodies:
Rabbit monoclonal to Cytokeratin 5 (clone EP1601Y), unconjugated Abcam Cat# ab52635, RRID:AB_869890
Rabbit monoclonal to Cytokeratin 5 (clone EP1601Y), AF 647 Abcam Cat# ab193895, RRID:AB_2728796
Rabbit polyclonal to Cytokeratin 5 (clone Poly19055), unconjugated BioLegend Cat# 905501, RRID:AB_2565050
Goat polyclonal Anti-Collagen Type I – 169Tm Standard BioTools Inc. Cat# 3169023D, RRID:AB_2810857
Rabbit polyclonal Anti-Collagen Type I, unconjugated Novus Biologicals Cat# NB600-408, RRID:AB_343276
Rabbit polyclonal Anti-Collagen Type III alpha 1, unconjugated Novus Biologicals Cat# NB600-594, RRID:AB_530879
Rabbit polyclonal Anti-Collagen Type IV, unconjugated Novus Biologicals Cat# NB120-6586, RRID:AB_789360
Recombinant rabbit monoclonal Anti-versican antibody (clone EPR12277), unconjugated Abcam Cat# ab240200
Rabbit polyclonal Anti-fibronectin, unconjugated Abcam Cat# ab2413, RRID:AB_2262874
Mouse monoclonal Anti-fibronectin (clone FN-15), unconjugated Sigma- Aldrich Cat# F7387, RRID:AB_476988

Mouse monoclonal to alpha-smooth muscle actin (clone 1A4), Cy3 Sigma- Aldrich Cat# C6198, RRID:AB_476856
Mouse anti-Histone H3 (C-terminus) (clone 1B1-B2), AF 594 BioLegend Cat# 819405, RRID:AB_2715800
Rabbit monoclonal Anti-p63 (clone EPR5701), unconjugated Abcam Cat# ab124762, RRID:AB_10971840
Mouse monoclonal Anti-human CD326/ EpCAM (clone 9C4), AF 488 BioLegend Cat# 324209, RRID:AB_756083
Mouse monoclonal Anti-human CD45 (clone H130), BV 605 BioLegend Cat# 304042, RRID:AB_2562106
Rabbit monoclonal Anti-fibronectin (clone EPR23110-46), 175 Lu, Standard BioTools Inc., Cat# 91H034175 RRID:AB_2927764
Anti-Vimentin (D21H3) – 143Nd, Standard BioTools Inc., Cat# 3143027D

Secondary antibodies:
Goat anti-rabbit IgG (H+L), AF 680 ThermoFisher Cat# 10585543
Goat anti-mouse IgG (H+L), DyLight 800 4X PEG Conjugate Cell Signalling Cat# 5257
Goat anti-rabbit IgG, DyLight 488 Invitrogen Cat# 35552
Goat anti-rabbit IgG, AF 647 ThermoFisher Cat# A-21244
Goat anti-mouse IgG, AF 546 Invitrogen Cat# A-11030
Goat anti-mouse IgG, AF 568 Invitrogen Cat# A-11004

Validation

All antibodies were manufacturer validated and commercially available. Validation statements are available on the manufacturer website.

Eukaryotic cell lines

Policy information about cell lines and Sex and Gender in Research

Cell line source(s)

We used primary human bronchial epithelial cells derived from airway brushings, and lung fibroblasts derived from lung tissue specimens from patients with IPF and controls.

Authentication

The cells were not authenticated.

Mycoplasma contamination

The cells were not routinely tested for mycoplasma. They were collected from subjects undergoing bronchoscopy who had samples sent for routine microbiological testing as part of standard clinical care, which were negative. Primocin, a broad-spectrum antimicrobial agent with activity against mycoplasma, was used in the cell culture media.

Commonly misidentified lines
(See ICLAC register)

There were no commonly misidentified cell lines utilized in this study.

Flow Cytometry

Plots

Confirm that:

☒ The axis labels state the marker and fluorochrome used (e.g. CD4-FITC).

☒ The axis scales are clearly visible. Include numbers along axes only for bottom left plot of group (a 'group' is an analysis of identical markers).

☒ All plots are contour plots with outliers or pseudocolor plots.

☒ A numerical value for number of cells or percentage (with statistics) is provided.

Methodology

Sample preparation

Cultured primary airway epithelial cells (passage #3) were plated in a 96-well round bottomed plate (2 x 105/ well). Cells were washed in PBS (Gibco), centrifuged (500xg, 4oC, 5 minutes) and the pellet resuspended in LIVE/DEAD fixable near-IR dead cell stain (1:1000 dilution in PBS; #L10119; Invitrogen). After a 20-minute incubation in the dark at room temperature, excess viability dye was removed with a PBS wash. Intracellular fixation and permeabilization buffer (eBioscience) was added to the cells overnight at 4oC in the dark. Cells were washed in PBS and stained with 150 µl unconjugated primary antibody anti-p63 and Human Fc block (1:50; BD Pharmingen) diluted in permeabilization buffer (eBioscience) for 30 minutes at 4oC in the dark. Cells were washed in permeabilization buffer and centrifuged. Secondary antibody staining was performed with Goat Anti-Rabbit IgG conjugated to BV421 (1:200; BD) diluted with permeabilization buffer in 2% BSA. Cells were washed and stained with remaining conjugated primary antibodies to EpCAM/ CD326, CD45 and KRT5 diluted in permeabilization buffer for 30 minutes at 4oC in the dark. After a final washing step, the cells were resuspended in FACS buffer.

Instrument

BD LSR Fortessa III

Software

FlowJo v7 (BD Life Sciences)

Cell population abundance

The samples analysed were passaged primary epithelial cells from submerged culture. Flow cytometry confirmed 100% of EPCAM+CD45- cells were KRT5 positive and 87% were TP63 positive. This is shown in Extended data Fig. 5a and 5b.

Gating strategy

Gating strategy are indicated in Fig. 3a. All gating strategy followed the same pattern: singlets (FSC-A/FSC-H), debris exclusion, removal of dead cells (negativity for viability marker), cells that were negative for CD45 but positive for EpCAM, then positive for KRT5 or p63. Fluorescence-minus one (FMO) controls were used to define the "positive" and "negative" population boundaries.

☒ Tick this box to confirm that a figure exemplifying the gating strategy is provided in the Supplementary Information.
